# Clinical characteristics of nontuberculous mycobacterial disease in people living with HIV/AIDS in South Korea: A multi-center, retrospective study

**DOI:** 10.1371/journal.pone.0276484

**Published:** 2022-11-10

**Authors:** Eun Hwa Lee, BumSik Chin, Young Keun Kim, Jin Sae Yoo, Young-Hwa Choi, Subin Kim, Ki Hyun Lee, Se Ju Lee, Jinnam Kim, Yae Jee Baek, Jung Ho Kim, Jin Young Ahn, Su Jin Jeong, Nam Su Ku, Joon-Sup Yeom, Jun Yong Choi

**Affiliations:** 1 Department of Internal Medicine and AIDS Research Institute, Severance Hospital, Yonsei University College of Medicine, Seoul, Republic of Korea; 2 Center for Infectious Diseases, National Medical Center, Seoul, Republic of Korea; 3 Department of Internal Medicine, Yonsei University Wonju College of Medicine, Wonju, Gangwon, Republic of Korea; 4 Department of Infectious Diseases, Ajou University School of Medicine, Suwon, Republic of Korea; 5 Department of Infectious Diseases, Gangnam Severance Hospital, Yonsei University College of Medicine, Seoul, Republic of Korea; Mie University Hospital: Mie Daigaku Igakubu Fuzoku Byoin, JAPAN

## Abstract

With the introduction of combination antiretroviral therapy (cART), the prevalence of human immunodeficiency virus (HIV)-associated nontuberculous mycobacteria (NTM) disease has declined. However, NTM diseases still occur in people living with HIV/acquired immunodeficiency syndrome (AIDS) (PLWHA). We analysed the clinical and microbiological features of NTM diseases in PLWHA in South Korea. PLWHA who were diagnosed with NTM diseases between January 2000 and March 2021 were retrospectively enrolled from five different hospitals in South Korea. Data on baseline demographics, HIV status, CD4+ T cell counts, viral load, past and current cART regimens, isolated NTM species, results of antimicrobial susceptibility tests, treatment regimens, and outcomes were collected by reviewing medical records. A total of 34 cases of NTM in PLWHA were included. Pulmonary and extrapulmonary NTM diseases accounted for 58.8% (n = 20) and 41.2% (n = 14), respectively. The lymph node was the most common site of extrapulmonary NTM disease (64.3%). The age at the time of NTM disease diagnosis was younger in the extrapulmonary NTM group than in the pulmonary NTM group (37.0 vs. 49.0 years). Mean CD4+ T cell counts at the time of NTM disease diagnosis was 186.6 cells/μL (range: 1–1394). Nine patients (26.5%) had fully suppressed viral loads at the time of NTM disease diagnosis. *Mycobacterium avium* complex (MAC) was the most common species found, followed by *M*. *intracellulare* and *M*. *kansasii*. MAC isolates were all susceptible to clarithromycin, but the rates of non-susceptibility to moxifloxacin, linezolid, ethambutol, and rifampin were 75%, 37.5%, 12.5%, and 12.5%, respectively. The average duration of treatment was 17 months and the mortality rate was 8.8%. NTM diseases may occur in PLWHA, even with completely suppressed viral loads. The identified clinical features of NTM diseases are essential for its clinical management in South Korea.

## Introduction

Nontuberculous mycobacteria (NTM) are free-living, saprophytic organisms that are ubiquitously present in soil and water [1]. NTM belong to the genus *Mycobacterium*, that includes *Mycobacterium tuberculosis* and *Mycobacterium leprae* [2]. Although NTM have low pathogenicity, they may become a pathogen for immunocompromised hosts such as people living with human immunodeficiency virus (HIV)/acquired immunodeficiency syndrome (AIDS) (PLWHA). The prevalence of pulmonary disease due to NTM is rising in countries such as the United States and South Korea [3, 4]. NTM may also manifest as extrapulmonary lesions in sites such as the lymph nodes and gastrointestinal tracts [5]. Before the introduction of combination antiretroviral therapy (cART), up to 43% of patients with AIDS acquired NTM infection [4]. In the 1980s, disseminated NTM disease was frequently found due to opportunistic infections in PLWHA [6]. A study from the United States (US) showed that the *Mycobacterium avium* complex (MAC) was the most common pathogen (61%), followed by *Mycobacterium fortuitum* (19%) and *Mycobacterium kansasii* (10%) in PLWHA [7]. For treatment of pulmonary and extrapulmonary MAC infections that exhibit susceptibility to macrolides, a 3-drug regimen including macrolide is recommended. Commonly, azithromycin (or clarithromycin) with rifampicin or rifabutin and ethambutol are used. Since epidemiologic studies are insufficient, no optimal treatment regimens, duration of therapy, and the role of surgery are clearly defined for most NTM species [8]. With the introduction of cART, the incidence of NTM infection in PLWHA has declined remarkably. However, we still encounter NTM infections in HIV clinics, especially in patients with advanced immunosuppression and poor adherence to cART [6].

Only few recent studies have analysed the epidemiology and clinical characteristics of NTM disease in PLWHA. In 2006, Jones et al. [6] discussed its clinical manifestations, diagnosis, treatment, and prophylaxis. In a prospective study, Miguez-Burbano et al. determined the epidemiology of NTM disease in PLWHA who required hospitalization [7]. The general epidemiology of NTM disease in PLWHA in Southeast Asia was analysed in 2012 and Oregon, USA in 2017 [9, 10].

In the modern cART era, the prevalence of opportunistic infection by MAC was <2 cases per 1,000 person-year [9–13]. The prevalence of NTM infection in Southeast Asian countries was 2% (19 of 1060) and none of the patients with HIV receiving cART developed NTM disease [9]. *M*. *abscessus* and *M*. *kansasii* were commonly found NTM pathogens [9]. In Oregon, USA, the median incidence was 110/100,000 HIV person-years with *M*. *aubagnense*, *M*. *avium*, and *M*. *chelonae* being the most common pathogens. In Africa, 247 out of 3068 patients with HIV were identified with NTM disease. *M*. *intracellulare* was the most common pathogen (47.8%), followed by *M*. *malmoense* (3.9%) and MAC (2.2%) [14]. In South Korea, two NTM cases were reported in 1,086 HIV/AIDS cohorts enrolled from December 2006 to July 2013 [15].

However, the latest studies lack the epidemiology and clinical characteristics of NTM disease, including both disseminated and non-disseminated forms. In this study, we analysed data from 34 NTM cases in five hospitals in South Korea from January 2000 to March 2021. We present an update on epidemiology, clinical characteristics, microbiological features, antibiotic sensitivity of NTM disease in PLWHA in South Korea.

## Methods

### Study subjects

This study protocol was approved by the Institutional Review Board of Severance Hospital, Yonsei University Health System, Seoul, Korea. (IRB No. 4-2021-0293). The requirement of written informed consent was waived due to the retrospective nature of the study.

### Study design and patients

In this retrospective study, we obtained the medical information of patients with a confirmed HIV diagnosis and NTM disease from January 2000 to March 2021. The data was collected and organized from March through April of 2021. Five different medical centers in South Korea participated in the study: Severance Hospital, a 2000-bed tertiary care teaching hospital; Gangnam Severance Hospital, an 824-bed tertiary care hospital; Ajou University Hospital, a 1200-bed tertiary care teaching hospital; Wonju University Hospital, an 866-bed tertiary care teaching hospital; National Medical Center (NMC), a 569-bed hospital. We excluded patients with an established diagnosis of NTM disease before HIV diagnosis.

We analysed all the specimens which were tested positive for NTM. The specimens were obtained from lymph node biopsy, respiratory samples such as sputum culture and bronchoalveolar lavage, pleural fluid, epidural abscess, intestinal lymph node and sigmoid biopsy. Identification of NTM and susceptibility testing are done at the Korean Institute of Tuberculosis (KIT). All culture and antibiotics sensitivity testing follows guidelines from Clinical and Laboratory Standards Institute (CLSI) [16].

### Diagnostic criteria for NTM disease

NTM infection and colonization were defined according to the 2007 American Thoracic Society (ATS)/ Infectious Disease Society of America (IDSA) [17]. The diagnosis of pulmonary NTM (p-NTM) disease required three components: respiratory symptoms, changes on chest radiography or computed tomography (CT), and isolation of NTM from bronchial lavage or at least two separate sputum samples. Patients who did not develop clinical symptoms or showed changes on imaging were excluded from the study. Pulmonary NTM was diagnosed if clinical and microbiologic criteria were satisfied according to the 2007 ATS/IDSA guideline [16]. Extrapulmonary NTM (ep-NTM) disease was defined as an NTM infection involving skin and soft tissue, lymph nodes, urine, blood, spinal fluid, or other normally sterile sites [10]. Disseminated NTM disease is defined as NTM infection involving more than one organ or positive result of blood culture or bone marrow [17, 18].

### Microbiological analysis

Respiratory specimens, including sputum and bronchoalveolar lavage samples, were processed and stained following ATS/IDSA guidelines [17]. The respiratory samples were decontaminated with 4% NaOH, homogenised, and centrifuged. Specimens were cultured in both 3% Ogawa medium and mycobacteria growth indicator tube medium (Becton Dickinson, NJ, USA) and observed until 8 and 6 weeks, respectively [19]. Identification of NTM and susceptibility testing are done at the Korean Institute of Tuberculosis (KIT). KIT is a supranational TB reference laboratory designated by WHO and International Union Against Tuberculosis and Lung Disease. For the identification of NTM species, polymerase chain reaction (PCR) and a restrictive fragment length polymorphism method based on the rpo B gene were used [20]. For the anti-mycobacterial susceptibility test, the broth microdilution method was used to determine the minimal inhibitory concentrations (MIC) of oral antibiotics (clarithromycin, ciprofloxacin, doxycycline) and parenteral antibiotics (amikacin, cefoxitin, imipenem). The results were interpreted according to the Clinical and Laboratory Standards Institute (CLSI) document M24-A in 2003 [16]. The anti-mycobacterial agents and susceptibility breakpoints are as follows. The MIC breakpoints in μg/ml for resistance was ≥32 for clarithromycin, ≥4 for ethambutol, ≥2 for rifampin, ≥1 for isoniazid, ≥4 for moxifloxacin, ≥32 for linezolid, and ≥2 for P-aminosalicyclic acid.

### Data collection

Information on age, sex, initial CD4+ T cell counts, viral load, past and current cART regimens, isolated NTM species, antimicrobial susceptibility test results, NTM treatment regimens, and outcomes were collected by reviewing electronic medical records. cART in this study was defined as a combination of two or more classes of antiretrovirals. HIV diagnosis and AIDS were defined according to the World Health Organization [21]. NTM isolated from any type of specimen, including sputum, lymph nodes, blood, bone marrow, cerebrospinal fluid, and synovial fluid were included in the study. The duration of hospitalization, mortality and the success of NTM treatment was also evaluated. CD4+ T cell count and viral load within 3 months from HIV diagnosis and NTM diagnosis were also collected. Treatment outcomes were categorised as treatment success, in treatment, and failure. The success of treatment was defined as the fulfilment of all of the following criteria: 1) clinical improvement; 2) medication for at least 6 months; 3) physician’s decision on completion of medication and treatment [22]. Treatment failure was defined as mortality or no clinical improvement with appropriate management for at least 6 months of treatment.

### Statistical analysis

Statistical analysis was performed using the Statistical Package for Social Sciences ver.26 (SPSS, Chicago, IL). Mann-Whitney’s U test and Fisher’s exact test were used for comparing pulmonary and extrapulmonary groups. Statistical significance was set at P<0.05.

## Results

### Demographic and clinical characteristics

A total of 51 PLWHA were reported to have NTM isolated from sputum (n = 35), lymph nodes (n = 12), gastrointestinal biopsy sample (n = 1), pleural fluid (n = 1), and epidural abscess (n = 1). However, 17 patients who had NTM isolated from one sputum culture but no related symptoms or change in radiographs were excluded from the study since they did not meet ATS criteria. Finally, 34 patients were included in the study.

Table 1 shows the baseline information of the patients. Most patients (86.3%) were male, and all were classified as Koreans. The average age at the time of HIV diagnosis was 41.6 years, and the average CD4+ T cell count and HIV viral load at initial HIV diagnosis were 80.7 cells/mm^3^ and 631.6×10^3^ copies/mL, respectively (Table 1). About 91.2% of patients had AIDS status at least once after being diagnosed with HIV. The most common cART regimen was the protease inhibitor (PI)-based regimen (41.2%), followed by the integrase strand transfer inhibitor (INSTI)-based regimen (39.2%). The ep-NTM group was diagnosed with HIV at a younger age than the p-NTM group (35.9 vs. 45.8 years, P = 0.013). Initial CD4+ T cell count at HIV diagnosis was lower in the ep-NTM group than in the p-NTM group, but the difference was not statistically significant.

**Table 1:** Demographic and clinical characteristics at HIV diagnosis.

	Total (n = 34)	Pulmonary NTM (n = 20)	Extrapulmonary NTM (n = 14)	P-value
Sex				
Male	29 (85.3%)	17 (85%)	12 (85.7%)	1.000
Female	5 (13.7%)	3 (15%)	2 (14.3%)	1.000
Age at HIV diagnosis (mean±SD year)	41.6± 11.5	45.8±12.2	35.9±8.2	0.015
CD4+ T cell count at initial HIV diagnosis (mean±SD cells/mm^3^)	80.7±114.5	108.0±129.7	47.0± 91.5	0.167
Viral load at initial HIV diagnosis (mean copies/mL)	631.6×10^3^	614.2×10^3^	651.6×10^3^	0.254
AIDS status, ever	31 (91.2%)	18 (90%)	13 (92.9%)	1.000
Initial cART regimen				
NRTI-based	1 (2.0%)	1 (5%)	0	
NNRTI-based	1 (3.9%)	0	1 (7.1%)	
INSTI-based	14 (39.2%)	6 (30%)	8 (57.1%)	
PI-based	15 (41.2%)	11(55%)	4 (28.6%)	
Unknown	6 (11.8%)	2 (10%)	1 (7.1%)	

AIDS, acquired immune deficiency syndrome; cART, combination antiretroviral therapy; HIV, human immunodeficiency virus; INSTI, integrase strand transfer inhibitor; NNRTI, non-nucleoside reverse transcriptase inhibitor; NRTI, nucleoside/nucleotide reverse transcriptase inhibitors; PI, protease inhibitor.

Values are expressed as the number of patients (%), unless otherwise described.

Table 2 presents a comparison of the clinical characteristics of patients at the time of NTM disease diagnosis. The average patient age at the time of NTM disease diagnosis was 44.1 years. The average age at diagnosis of the ep-NTM group was lower than that of the p-NTM group (37.0 vs. 49.0 years, P = 0.013). The p-NTM group had a lower body mass index (BMI) at NTM diagnosis than the ep-NTM group (P = 0.010). The average time from HIV diagnosis to NTM diagnosis was 29.7 months. The ep-NTM group appeared to have shorter time intervals between HIV and NTM diagnosis than the p-NTM group, but the difference was not statistically significant. The number of patients with suppressed viral load, to an undetectable range, was 7 out of 20 patients (35.0%) and 2 out of 14 patients (14.3%) in the pulmonary and extrapulmonary NTM groups, respectively. Most patients were receiving cART at the time of NTM diagnosis (75% for the p-NTM group and 78.5% for the ep-NTM group).

**Table 2:** Demographic and clinical characteristics at NTM diagnosis (n = 51).

	Total (n = 34)	Pulmonary NTM (n = 20)	Extrapulmonary NTM (n = 14)	P-value
Age at NTM diagnosis (mean±SD year)	44.1± 12.7	49.0± 13.9	37.0±7.9	0.007
BMI at NTM diagnosis (mean±SD kg/m^2^)	19.3±2.5	17.9±2.2	21.1±1.6	0.007
Time from HIV diagnosis to NTM (mean±SD months)	29.7±47.3	38.3±54.0	10.8±25.2	0.377
CD4+ T cell count at NTM diagnosis (mean±SD cells/mm^3^)	186.6±273.7	181.0±181.2	220.9±413.5	0.373
Viral load at NTM diagnosis (copies/mL)	631.6×10^3^	928.3×10^3^	240.1×10^3^	0.830
Patients with suppressed viral load at NTM diagnosis	9 (26.5%)	7(35.0%)	2(14.3%)	0.249
On cART at NTM diagnosis				
Yes	26 (76.5%)	15 (75%)	11 (78.6%)	
No	6 (17.6%)	3 (15%)	3 (21.4%)	
Unknown	2 (5.9%)	2 (10%)	0	
Type of ART regimen at NTM diagnosis				
NRTI-based	1 (1.9%)	0	0	
NNRTI-based	2 (3.9%)	0	0	
INSTI based	20 (39.2%)	11 (55%)	8 (57.1%)	
PI-based	21 (41.2%)	6 (30%	4 (28.6%)	
INSTI+PI	1 (1.9%)	1 (5%)	0	
Unknown	6 (11.8%)	2 (10%)	1 (7.1%)	
Not on ART	0 (0.0%)	0	1 (7.1%)^†^	
Duration of NTM treatment (mean±SD months)	14.2±9.1	15.4±8.4	17.0±8.9	

^†^ cART discontinued due to immune reconstitution inflammatory syndrome (IRIS).

AIDS, acquired immune deficiency syndrome; cART, combination antiretroviral therapy; HIV, human immunodeficiency virus; INSTI, integrase strand transfer inhibitor; NNRTI, non-nucleoside reverse transcriptase inhibitor; NRTI, nucleoside/nucleotide reverse transcriptase inhibitors; PI, protease inhibitor.

Values are expressed as the number of patients (%), unless otherwise described.

NTM disease was classified as pulmonary or extrapulmonary depending on the site of the lesion. Of the 34 NTM disease cases, 20 (58.8%) were considered p-NTM disease and 14 (41.2%) were ep-NTM disease. The classification of the different NTM disease groups is displayed in Table 3. The lymph node was the most common site of ep-NTM disease. The single intestinal NTM case was diagnosed using sigmoid biopsy.

**Table 3:** Type of NTM disease and site of infection.

Site of infection	Number of patients (%)
Pulmonary	**20 (58.8%)**
Extrapulmonary	**14 (41.2%)**
Lymph node	12 (35.3%)
Gastrointestinal tract	1 (2.9%)
Epidural space	1 (2.9%)
Disseminated	2 (5.9%)

NTM, nontuberculous mycobacterium.

### Microbiological characteristics

The most common NTM species isolated from the patients was the *Mycobacterium avium* (Table 4). The other species commonly found were *Mycobacterium intracellulare* (six cases) and *Mycobacterium kansasii* (three cases). The types of NTM isolated from pulmonary and extrapulmonary NTM disease were similar, with *M*. *avium* being the most common pathogen, followed by *M*. *intracellulare and M*. *kansasii*. In one case in the p-NTM group, a mixed infection of *M*. *avium* and *M*. *intracellulare* was identified.

**Table 4:** Type NTM species isolated.

NTM species	Total (n = 51)	Pulmonary NTM (n = 20)	Extrapulmonary NTM (n = 14)
*Mycobacterium avium*	19	11	8
*Mycobacterium intracellulare*	6	3	3
*Mycobacterium kansasii*	3	2	1
*Mycobacterium chelonae*	1	1	0
*Mycobacterium fortuitum*	1	1	0
*Mycobacterium gordonae*	1	1	0
*Mycobacterium celatum*	1	1	0
Mixed infection	1	1^†^	0
Unidentified	7	2	5

^†^ Mixed infection.

NTM, Nontuberculous mycobacterium.

Values are expressed as the number of patients.

Once NTM was isolated from the culture, an antibiotic susceptibility test was performed in 14 out of 34 samples. Due to test availability in different centers, not all samples were tested for drug susceptibility. Table 5 shows the percentage of antibiotic resistance of each pathogen to the antibiotics commonly used in NTM treatment. The intermediate response was considered positive resistance to antibiotics. MAC was susceptible to clarithromycin, but mostly resistant to moxifloxacin, linezolid, ethambutol, and rifampin. *M*. *intracellulare* was susceptible to clarithromycin, but resistant to moxifloxacin and linezolid. *M*. *kansasii* showed resistance to isoniazid and p-aminosalicylate.

**Table 5:** Antibiotics resistance.

Type of organism	No. of cases	CLA	ETM	RIF	INH	MOX	LIN	PA
MIC breakpoint for resistance (μg/ml)		≥32	≥4	≥2	≥1	≥4	≥32	≥2
*M*. *avium*	8	0/8	1/8	1/8	0/8	6/8	3/8	Not identified
		(0%)	(12.5%)	(12.5%)	(0%)	(75%)	(37.5%)	
*M*. *intracellulare*	6	0/6	0/6	0/6	0/6	6/6	6/6	Not identified
		(0%)	(0%)	(0%)	(0%)	(100%)	(100%)	
*M*. *kansasii*^†^	1	0/1	0/1	0/1	1/1	0/1	0/1	1/1
		(0%)	(0%)	(0%)	(100%)	(0%)	(0%)	(100%)

^†^ One case was a mixed infection of *M*. *avium* and *M*. *intracellulare*.

CLA, clarithromycin; ETM, ethambutol; RIF, rifampin; INH, isoniazid; MOX, moxifloxacin; LIN, linezolid; PA, p-aminosalicylate.

### Treatment outcomes

The average duration of treatment in patients who completed treatment was 16 months and 19 months for the pulmonary and extrapulmonary NTM disease groups, respectively. Treatment outcomes were categorised into treatment success, in treatment, and treatment failure. In total, 41.2% of patients completed the treatment successfully. The p-NTM group had a treatment failure rate of 20%, which was higher than the failure rate in the extrapulmonary group (14.3%). In most cases (21 out of 34), the reason for rehospitalization was to perform diagnostic exams such as lymph node biopsy and bronchoscopy with bronchial lavage. The mortality rate in the p-NTM group and ep-NTM group was approximately 10% and 7.1%, respectively (Table 6).

**Table 6:** Treatment outcomes of NTM.

	Treatment duration (months)	Treatment outcome			Hospitalization	Mortality
		Treatment success	In treatment	Treatment failure		
Total (n = 34)	17	14 (41.2%)	14 (41.2%)	5 (14.6)	22 (64.7%)	3 (8.8%)
Pulmonary NTM (n = 20)	16	6 (30%)	10 (50%)	4 (20%)	11 (55%)	2 (10%)
Extrapulmonary NTM (n = 14)	19	7 (50%)	5 (35.7)	2 (14.3)	10 (71.4%)	1 (7.1%)

NTM, Nontuberculous mycobacterium.

## Discussion

The distribution of NTM reflects geographical diversity, wherein species vary according to region and country [23]. According to an epidemiological report on NTM in South Korea, MAC followed by *M*. *abscessus* is the most common pathogen implicated in p-NTM disease. In contrast, *M*. *kansasii* rarely causes the disease [23]. In ep-NTM disease, the common pathogens are *M*. *intracellulare* (38.9%), followed by *M*. *avium* (23.1%), and *M*. *abscessus* (8.4%) [24]. In our study, the most common pathogen for pulmonary and extrapulmonary NTM disease was *M*. *avium*, followed by *M*. *intracellulare* and *M*. *kansasii*. Interestingly, although *M*. *kansasii* is an uncommon pathogen in immunocompetent hosts, it can be an important pathogen in PLWHA.

Risk factors associated with pulmonary NTM disease include older age, bronchiectasis, chronic obstructive pulmonary disease (COPD) for non-HIV population [25]. While NTM affects mostly older women in the general population, young men are predominantly affected in the HIV population. The percentage of females with NTM disease in the general population was 54.6% and prevalence was high in women aged ≥50 years, with a peak at 70 years (223.0 cases/100,000 population) [4]. In our study population of PLWHA, men were predominantly affected (85.3%) and the average age was 41.6 years.

Extrapulmonary involvement of NTM includes lymphadenitis, skin disease, and a disseminated form. Lymphadenitis usually involves a single site, typically the submandibular and cervical area and less commonly the axillary and inguinal sites. Skin diseases manifest as nodules, subcutaneous abscesses, pustules, and ulcers. The disseminated form is found in patients with immunocompromised conditions, such as HIV/AIDS, hematologic malignancies, treatment with immunosuppressive agents, and solid organ transplantation [5]. In our study, extrapulmonary NTM disease was diagnosed at a younger age and had a higher HIV PCR titre than the p-NTM group. This finding emphasises the importance of performing a precise and detailed initial examination, especially for young patients with HIV who presented with low CD4+ count and high viral load at initial HIV diagnosis.

According to the guidelines for opportunistic infection of HIV by the National Institute of Health (NIH), MAC typically occurs in PLWHA with CD4+ T cell count <50 cells/mm^3^ and plasma HIV RNA level >1,000 copies/mL [26]. The NIH guidelines do not recommend antibiotics as NTM prophylaxis for PLWHA who start cART. However, the NIH guidelines recommend NTM prophylaxis for patients who remain viraemic and with a CD4+ T cell count <50 cells/mm^3^, with no option for ART for full suppression of virus [24]. In our study, the average CD4+ T cell count and viral load in patients diagnosed with NTM were 190.7±264.1 cells/mm^3^ and 631.6×10^3^ copies/mL; 76.5% of the patients were receiving appropriate ART. Our study suggests that PLWHA with CD4+ T cell count >50 cells/mm^3^ and are receiving cART are not immune from acquiring NTM disease. Successful viral suppression was not found to guarantee complete protection from NTM disease. Approximately 35.0% and 14.3% patients in the pulmonary NTM and the extrapulmonary group, respectively, showed viral suppression to the undetectable range but acquired NTM disease.

In our study, 20 out of 34 patients were diagnosed with NTM disease within 6 months from HIV diagnosis, and 7 out of 34 cases were diagnosed within 1 month. Therefore, evaluation for NTM disease at the initial clinical evaluation is essential for early diagnosis and treatment. For people with newly diagnosed HIV and low CD4+ T cell count, an initial sputum culture and chest radiography may be helpful in detecting pulmonary NTM disease. In addition, the presence of lymphadenopathy should be carefully examined to evaluate for extrapulmonary NTM disease.

The introduction of cART significantly reduced the incidence of NTM disease in PLWHA [7, 27]. NTM disease incidence in the HIV-positive group decreased from 2.29 to 0.07 events per 1000 patients from 1997 to 2010 [27]. In our study, six cases of NTM disease were diagnosed before 2011 and 25 cases were diagnosed in 2011–2021. Although the study population was small, this trend indicated the persistence of NTM disease even in the modern cART era.

The incidence of NTM disease is increasing in developed countries in non-HIV populations. From 1997 to 2010, the incidence of NTM diseases in the HIV-negative group increased from 2.91 to 3.97 events per 1,000,000 patients [27]. An increase in NTM disease in the general population may impact immunocompromised populations, including PLWHA. Additionally, the prevalence may be re-evaluated after the introduction of the INSTI in 2007.

## Conclusions

PLWHA are vulnerable to various opportunistic infections, especially during the initial phase of cART, when CD4+ T cell count <200/mm^3^. Therefore, such immunocompromised patients should be carefully evaluated for opportunistic infections. NTM is a common opportunistic infection in PLWHA. The pulmonary system and lymph nodes are the most common sites of NTM disease. MAC is the most common NTM followed by *M*. *intracellulare* and *M*. *kansasii*. A CD4+ T cell count >50 cells/mm^3^, viral suppression, and treatment with cART do not guarantee protection from NTM disease. Therefore, continuous monitoring is required for early detection and treatment of the disease.

## Supporting information

S1 Data Clinical data without private information. (XLSX)

S1 Appendix (DOCX)
